# Who's choosing whom? A sociological study of the specialty choices in a Danish context

**DOI:** 10.5116/ijme.4dad.c33f

**Published:** 2011-04-27

**Authors:** Pernille Dehn, Berit Eika

**Affiliations:** Centre for Medical Education, Aarhus University, Denmark

**Keywords:** Specialty choice, career choice, medical sociology, theory-based approaches, Bourdieu’s conceptual framework

## Abstract

Objectives: The purpose of this study was to investigate if habitus, the unconscious and embodied mental structures founded early in life, can contribute to our understanding of how individuals choose a medical specialty.

**Methods:**

A qualitative approach was employed using standardized open-ended interviews. In the present research, sampling was purposive, with an aim to illuminating the study objective. A sample of six juniors and three senior doctors were recruited from gynecology and obstetrics, vascular surgery and general practice via a snowball method. The interview guide and the subsequent analysis were based on Bourdieu’s sociological theory.

**Results:**

Three central themes emerged, labeled as “the use of distinctions and dichotomies”, “the shaping of habitus” and “consequences of the shaping of habitus”. These represent values and preferences developed through childhood education and experiences which may contribute to explaining specialty choices. Participants distinguished between specialties by referring to dichotomous characteristics of the specialty (such as sick/healthy patients; young/elderly patients; fine/coarse surgery).

**Conclusions:**

Bourdieu’s theory is useful for broadening our understanding of specialty choice, as his central concept, habitus, was found to direct the choice of specialty and constrain the number of possible specialties for the individual doctor. Research is needed to better understand how various factors affect the specialty choices of medical school graduates.

## Introduction

Every specialty has a unique historicism. Accordingly, a specialty may be concerned with the same organ or patient group over time but also undergo changes in response to trends such as the development of new treatment modalities, the inclusion of newly defined disease entities, or changing political agendas. Consequently, the specialty's characteristics change and, as a result, the characteristics of the group of doctors who find the specialty attractive might also change.

In Denmark, gynecology & obstetrics, for example, is a specialty that has faced considerable changes over the past few decades because of a continuous sub-specialization, a stronger internal focus on research, and an increased focus on patient communication. These changes are reflected in the way the Danish Society of Obstetrics and Gynecology describes their field of work.^1^ Previously, the specialty was described as embracing specific organs and diseases. To-day, it is described as a specialty concerning “women” in a very broad sense of the term. Furthermore, in the same period, the specialty has changed from being male-dominated to becoming a popular choice for young female doctors.^2^

### Why another study on choice of specialties?

Choice of specialties has been studied from many different angles via different methodologies. The existing literature provides valuable knowledge about the complex and variant elements that together constitute the issue of specialty choice.

Many studies focus on particular elements, such as one specific specialty’s patterns of recruitment^3-4^ or the importance of role models; ^5-10^ while other studies consider personality perspectives, either within psychological models^11-13^ or by applying broader explanatory designs focusing on young doctors’ expectations of long-term lifestyle as a determinant of career choice.^14-16^

Two main reasons were determined for exploring the subject of specialty choice. Firstly, there are few studies in the literature that consider a broader timeframe by integrating influential aspects in the past, present and future as a whole. Secondly, much of the current knowledge on choice of specialty rests on the assumption that any action is preceded by rational thinking ^7, 17-19^ and as such, it may not provide a complete picture. By assuming that choice of specialty is rational, it is also assumed that this choice is exclusively conscious and deliberate. This assumption, however, does not take into consideration the influence of other potentially important determinants already embedded as personal characteristics of the individual.

More qualitative insight into the mechanisms involved might expand the perception of specialty choice as more than a merely rational decision. This study was inspired by career studies in other areas to consider new ways of examining young doctors’ choice of specialty.^20,21^ The aim was to reach an understanding of how both conscious and unconscious factors in combination outline ways of reasoning and acting. Bourdieu, the French philosopher and sociologist, provided the necessary theoretical framework to pursue this aim.

### Bourdieu’s conceptual framework

Bourdieu's theory allows for an exploration of the complex relations between social structures and “agents”, e.g. doctors, organizations and specialties (the latter as constructs representative of thinking and acting individuals). Bourdieu’s concepts, which were developed on the basis of empirical studies, create and investigate relational dynamics in a specific field (e.g., the medical world) and the dispositions and resources held by each agent that influences his or her positioning herein. Three concepts, habitus, field, and capital, are central for the understanding of Bourdieu's theory and will be briefly described in the following paragraphs.

Bourdieu's concept habitus encompasses the non-reflective, embodied mental structures directing our actions, practices and meaning. The structures are originated from socio-economic background, value systems, norms and cultural habits and represent specific ways of thinking and acting.^22^ Habitus is mainly founded in childhood through education but is restructured throughout life. It forms the basis of individual differences and equips the individual with specific dispositions for acting and attributing meaning to things and experiences.

Field is the context within which agents position them-selves.^23,24^ Within the medical field, the various specialties have employed different strategies to position themselves. As with any other field, the medical field has its own rules reflecting norms and values that are constantly being negotiated. Norms and values within a specialty might be a privilege, a technique, or specialized knowledge. The position of each particular agent in a field is a result of the interaction between the specific rules of the field, the agent's habitus, and the agent's resources or “capital.”^23, 24^

Capital is used here as an illustration for acknowledged values in the field, including economic capital, cultural capital (possessing the “correct” knowledge and taste), social capital (having the “right connections” and effective social skills) and symbolic capital (linked to status and prestige). These different forms of capital are converti-ble.^25^As an example, a senior doctor who possesses a PhD can use this cultural capital to gain access to a prestigious position as a professor thereby obtaining symbolic and economic capital. Testing the applicability of Bourdieu's theory to the choice of specialty thus represented an overall objective of the study.

### Objectives

The purpose of the study was two-fold. The first aim was to investigate the coherence between the shaping of the habitus and the choice of specialty; the second aim was to explore how characteristics of specific specialties influence the choice of specialty. Regarding these objectives, the study is an initial assessment of a small group of doctors in a limited number of specialties.

## Methods

The Danish six-year undergraduate medical program is followed by a one-year internship. The following specialty training consists of a one-year induction program followed by four to six years of specialist training, depending on the chosen specialty. Young doctors might have induction program employments in more than one specialty if they are in doubt about their specialty choice after the first induction year.The access to specialty training is regulated first by the National Health Authorities (NHA), who, together with the colleges of the different specialties, decides on the number of future specialists within each specialty. A specialty-specific employment committee assesses the applicants for the specialty. Thus, the choice of medical specialty is constrained not only by qualifications of the young doctor but to a certain extent by external structural factors as well.

### Participants

The informants were nine medical doctors employed in the Greater Copenhagen area. Three of the informants were from the field of gynecology & obstetrics, three were from vascular surgery, and three were from general practice. From each specialty, two doctors undergoing specialty training and one consultant in charge of such training were included. The specialties were selected for their diversity and unique characteristics: gynecology & obstetrics as a multi-faceted specialty treating a considerable proportion of healthy patients as well as critically ill cancer patients; vascular surgery as a specialized surgical field treating mainly elderly patients with specialized treatment modalities; and general practice as covering broader aspects of health care than the other two specialties. The integrated specialties differ furthermore with regard to a range of other features described and discussed in this article. In order to expose the specialties' historicism by identifying different inclusion and exclusion mechanisms over time, informants of different educational status and age were included.Inclusion criteria for senior doctors of the study population were the possession of formal educational obligations and at least ten years of practical experience as specialized doctors; junior doctors were required to have employment in either an induction training program or in specialist training. Informants were chosen as a purposeful sample;^26^ one doctor was selected from each of the three specialties fulfilling the stated criteria and contacted via email by the first author. Following the first round of interviews, using the snowball method, the remaining six informants were chosen for their perceived ability to elaborate or explicate issues that had already been introduced. Informants were two males and seven females, aged between 30 and 52.This study was exempt from ethical approval according to Danish law. Regardless, considerable effort was made to protect the interests of the informants; voluntary and informed consent was sought from all informants prior to their participation in the study, and transcriptions were coded to ensure confidentiality. The informants were additionally encouraged to contact the research team if they had any concerns or questions.

### Data Collection

The empirical material collected in 2007 comprises nine individual interviews, lasting approximately one hour each, performed by the first author on the basis of an interview guide. Seven interviews were conducted at the informants’ workplace, and the last two were conducted in the home of the informants.

The theoretical perspective of Bourdieu was pivotal to the current study and played an important part in the design of the study.^27^ The questions within the interview guide also concerned subjects that normally are not associated with choice of specialty. The informants were for example asked: Please tell me about your home as a child. This question aimed to obtain information about type of home (academic, artistic, craft) and how spare time was used for sport and activities (such as holidays) and was followed up by questions aiming to elaborate the first question. By using mostly open-ended questions and by encouraging the informants to exemplify their statements, the interviews generated detailed and rich descriptions of the informants' personal life experiences. These descriptions concerned such information as parents’ social status and employment, family life and habits, childhood experiences, leisure time, school, and social life. In order to uncover their experiences with different specialties the informants were asked for example: Please describe one or two experiences that have given you insight into other specialties (e.g., duties during your period as a student at medical school, clinical experiences, locum tenancy). What happened?

Data obtained from the interviews were supplemented with studies of the formal descriptions of the specialist training courses, including national learning objectives for the included specialties, to unfold the specialties' own articulation of their characteristics (triangulation).^2,26^ Interviews and descriptions mutually inspired each other, thereby providing additional perspectives and validation.

The interviews were recorded, transcribed verbatim, and depersonalized. All informants were given the opportunity to comment on the transcribed interviews to achieve a fully transparent process.

### Data Analysis

The analysis was performed as described by Kvale.^28^ Categories for the specialty choices were already defined in the interview guide. From the categories, “back-ground/habitus”, “medical experiences/process of choice”, “construction of the specialty”, “relations in the specialty”, and “inclusion/exclusion”, values and norms, family habits and taste, education and experiences shaping the life of the informants were identified.As a next step, the informants’ combinations of capital were identified within the categories by critically describing resources and experiences that had provided them with skills, knowledge and preparedness. From these pictures constituted by the different combinations of capital, it was possible to determine the informant’s habitus and dispositions in relation to positioning themselves in the medical world. Different values, norms and preferences, as perceived by the informants within the specialties, were compared to those expressed through the habitus of the informants. Similarities and differences were determined and interpreted by both authors.The analysis thereby allowed for an understanding and an interpretation of coherence between the process of shaping the habitus and the structural constraints met along with the individual’s latitude to choose a career.

## Results

### The use of distinctions and dichotomies

The informants’ descriptions of what characterized their specialty were widely based on distinguishing features. Informants would describe valued actions, procedures or behavior to pinpoint the essence of the specialty, but they would also distance and dissociate these descriptions from other actions, procedures and behavior that they found were in contrast to themselves. In the interviews, it was apparent that dichotomies serve to distinguish between the specialties. These related to patient type (sick versus healthy, young versus elderly) and primary treatment characteristics (short versus long term, conclusive versus inconclusive result). Furthermore, a number of more specific differences concerning social conventions and medical content were found. In reacting to experienced actions, values, norms and contexts, the informants described their experiences and thoughts in terms of non-identification as much as self-identification. The informants' descriptions of each specialty provided an image of their personal stance in relation to the described characteristics.

### The shaping of habitus – Informants’ norms and values

The socio-economic backgrounds of informants varied considerably and provided no independent explanation for choice of specialty. During their childhood, however, informants differed on a number of variables, including their focus on their schooling, their physical activity, their manual ability, their interest in natural sciences, and their tendency toward individualism or working cooperatively with others. Through their upbringing, they thus incorporated different norms and values. One of the informants, Anna, with a relatively privileged family background, tells about her path toward the medical field:

“…both my siblings have university degrees and essentially all my cousins too”

And she continues:

*“...it was a very academic home. And I never thought of alternatives to attending college. And subsequently, I have kind of been puzzled about this lack of reflection on other possibilities to pursue. But I have been kind of inculcated right from a very early stage of life that I would go to college and university”* (Anna, 33 years old, junior doctor in GP)

The data revealed that different family values and practices do produce different expectations, often not even articulated. This is also illustrated in the narrative of the informant Laura, depicted in the vignette (See Appendix 1). When she talks about her childhood home, she uses words like “respect” and “room for everyone” to describe the basic values she learned there.Several aspects from Laura's life may be seen as contributory factors to her choosing first to enter the medical field and then later to become a gynecologist specializing in cervical cancer. As a consequence of her position in the "social space" with her grandparents, who belonged to a working class culture, Laura developed a certain perspective on the importance of school and of her own role in society, e.g., that you need to do your homework and appreciate the abilities with which you have been endowed. They should not "go to waste," she says. Her sense of social responsibility, incorporated as a value in her childhood, directs her toward a position where she obtains the feeling of fulfilling this purpose. Her creative activities have given her the ability to create and express herself physically. This ability is among the reasons for Laura’s attraction to certain specialties which require surgical skills. After experiencing different specialties, she ended up limiting her choices to urology, gynecology and oncology – these specialties can therefore be considered as constituting Laura’s “space of possibility”. However, her interest in problems relating specifically to females and the appeal of working with colleagues who share her way of thinking are determinants that led her toward her final choice, gynecology. Even though they fulfill her need to achieve specific, visible results, and a sense of making a difference, the other specialties in her “space of possibility” do not provide as good a match to her personal values as gynecology does.Overall, differences were found regarding taught values, family practices and attitudes toward education, and social skills. Such overall trends were pronounced and occurred in various combinations. As such, these trends were seen to shape preferences, influence the focus of the person and, over time, change the habitus of the informants. In conclusion, the informants’ habitus caused them to either like or dislike and thereby include or exclude certain choices in life; it also provided them with different interpretations of basic phenomena.In the following, for each of the three specialties in the study, descriptions are provided to illustrate how specific values both shaped the choice of that specialty and disqualified others.

### Forming gynecologists’ choices: values and distinctions from other specialties

Among the informants from the gynecological specialty, an explicit stress on equality, empathy, and solidarity was detected. The three informants had early in their careers been involved in work reflecting such values: one had served as a volunteer in organizations working for women's rights, one had been involved in work on primitive third-world maternity wards, and the third had worked as a counselor at an organization aiming to help mothers and pregnant women. They all had a very distinct approach to their patients, as articulated here by Louise, an obstetric specialist and trainer:

*…“we are advocates for the women, who are our patients”…* (Louise, 52 years old, senior doctor, obstetric specialist)

The requirement to work in night shifts was, according to the informants, accepted due to the “acute” and “signifi-cant” nature of much of the work within their specialty, as opposed to night duties in internal medical specialties where patients are seldom in need of urgent medical care. Karen, a future gynecologist, exemplifies this when telling about her experiences from a medical specialty:

*…”it was meaningless to stay up until three at night to update a medical chart of an old lady that I had discharged from hospital two days earlier and now had come back because she felt she had respiratory problems”…*(Karen, 30 years old, junior doctor in gynecology)

In this way, the meaningfulness of certain aspects of a given specialty forms one of the parameters for choosing or rejecting it. In terms of habitus, it is clear that, for a person with a strong drive to bring about change, immediacy of impact plays an important role.The two informants who had entered the specialty more recently had acquired special competencies during their childhood or adolescence. Through intensive practicing of high level competitive sports, they had learned to work in a goal-oriented and structured manner. These competencies were appropriate in a specialty that, according to these informants, has a PhD degree as a de facto requirement.Unlike the two younger doctors, Louise, the senior doctor, had not experienced the research focus or the possession of a PhD as important inclusion criteria when she entered the specialty. She had been very active in developing educational and political issues within the specialty society. Thus, she engaged with her work in this way by performing an activity also regarded as valuable within the specialty.Finally, the gynecologists all stressed the opportunity to work within a wide range of fields within gynecology as opposed to a specialty with a specific, limited focus. They also appreciated working in an open learning environment versus a situation that requires extensive preparation in order to gain access to the knowledge of the more experienced doctors.

### Forming vascular surgeons’ choices: values and distinctions from other specialties

The informants from the specialty of vascular surgery valued close teamwork and emphasized visible results and continued recognition of the work done. It gave them great satisfaction when patients who had been admitted in poor conditions would, as a result of the treatment, get up and leave on their own accord.

The social conventions of the field of vascular surgery were, according to the informants, characterized by humor and room for diversity in comparison to their previous experiences.

The informants made an effort to distinguish vascular surgery from the fields of more general surgery. Cecilia, who is training for vascular surgery, uses distinctions to underpin her preference for her specialty:

*…”this specialty gave me some of the things that really interested me – the finer surgery, that is not the coarse surgery, and then I found it was an interesting specialty because our patients – there is also a large amount of medical issues in our specialty – it is not merely surgery”…* (Cecilia, 31 years old, Junior doctor in training for vascular surgery)

The vascular surgeons also distinguished their specialty from other specialties by eschewing stability in favor of new and exciting advanced technical treatment modalities, valuing a friendly versus unfriendly tone at the workplace, and embracing humor over seriousness. Descriptions of learning objectives made specific reference to the specialty’s developments as seen in an international context. Inclusion in the specialty was highly dependent on technical skills.

### Forming general practitioners’ choices: values and distinctions to other specialties

Family values, such as the importance of close relationships, were strong in all three representatives of general practice. Thus, family was a pivotal element in their upbringing. The informants agreed that general practice provided them with considerable autonomy and sufficient freedom to prioritize other facets of life than just being a doctor. This was expressed in different ways by the informants. For example, one commented that work as a GP was a natural extension of the freedom he had experienced while studying medicine; therefore, he had chosen general practice in order to be able to build his own career.Examining the norms and values of Anna, the young informant training for general practice, her habitus appeared to be influenced by strong social and cultural values; through her upbringing, she had learned to appreciate a gentle behavior and tone. A quotation from the interview with Anna traces a connection between her childhood and her preference regarding patient and staff relations:

*…”when I had my training program in general practice, I thought for the first time: Ooh, I really like it here. And I liked the procedures and the staff... […]..I think it is because when you enter general practice, it is such a small entity, and you have to be nice and polite to each other, and you have to have close communication”…*(Anna, 33 years old)

The overall impression among the general practice informants was that they valued the diversity they experienced in their daily work as opposed to boring routines. Also, the close relations to patients and staff alike differed from their experiences with patient and staff relations in hospital settings. General practice informants stressed a holistic view versus a mal-functioning apparatus approach. Inclusion and exclusion from this specialty were generally experienced as a question of liking or disliking the working conditions.

### Consequences of the shaping of habitus

Experiences and encounters which the informants found inconsistent with their self-image contributed to the shaping of habitus and helped to qualify their selection. As a consequence of the shaping of habitus, the actual choice of specialty of the informants was constrained and they ended up choosing from a limited subset of specialties. Within these few specialties, the informants identified recognizable and valued behavioral patterns similar to their own - not just as doctors but as individuals. Consequently, some specialties became entirely unthinkable as a choice of career.

## Discussion

The understanding of how informants perceive the characteristics of each specialty as distinct from the others is closely attached to their respective habitus. Thus, habitus serves to explain their choice. Options are produced from the sum of the various forms of capital and the specific combination of all embodied experiences, values and norms within each individual in conjunction with deliberated rational reflection and structural constraints. Specialty choice is thus not just about choosing but also very much about fitting in and being chosen by experienced specialists who find the young doctor to be talented. Specialty choice becomes a matter of recognizing shared values and ways of dealing with professional challenges. Within the three specialties studied, we found that the various characteristics related to knowledge, beliefs and behavior are presented and valued differently within each specialty.For the gynecological informants, other determinants for specialty choice included fundamental values and qualities such as solidarity and empathy – values they shared with doctors already working within the field. In a specialty like gynecology-obstetrics that currently enjoys a considerable influx of doctors wishing to enter the specialty and that is characterized by high research expectations, goal orientation is important in determining if the specialty represents a suitable option. Being goal-oriented and disciplined may therefore be interpreted as prerequisites to entry and success in this field. This goal orientation was precisely a characteristic of the younger informants, shaped by their engagement in high-level competitive sport in their adolescent period.One of the vascular surgery informants had been raised in a family running its own business. According to Bourdieu having a self-owned business as one’s economic foundation induces a certain mindset with a strong focus on initiative and achieving a benefit or result. The same general mindset is present when an individual is driven by a desire to achieve a specific, visible result, which is frequently the case when the objective is "making a difference" in the lives of other people. Bourdieu contends that such a goal-oriented mindset is internalized in the habitus and subconsciously transferred to other areas of life.^29,30^ This mindset is mirrored in the field of surgery, as treatments are characterized by an immediate improvement in the patient's condition. In a professional context, the possibility of achieving rapid, specific results is given considerable importance. This tendency was a clear guiding principle for informants choosing the specialty of vascular surgery. Other specialties within their “space of possibility” either comprised a great share of surgery, or social conventions and organization similar to those found in vascular surgery.The value of family is embedded in general practice and in the holistic perception of patients. As such, this specialty supports a well-known and treasured value, and its organization leaves room for several concurrent priorities. In addition, close, long-term relationships to patients and staff were an important consideration for informants to feel "welcomed" to the specialty.

### Perspectives

By examining how informants’ embodied habitus both attracts them to and makes them attractive to individuals with a similar habitus, the analysis shed light on how some specialties come to be seen as "good" options and others are excluded by junior doctors.The present study gives rise to rethinking the perception of choice of specialty and gives reason to include sociological perspectives in further studies on choice of specialty. The factors outlined herein should be included as a supplement to rational determinants. The study could also serve as a basis for action for helping young doctors determine their choice of specialty. By identifying common values and a shared professional profile of a specialty, it could be possible to highlight the specific profiles of the specialties in question. A more comprehensive description of each specialty may be used as a specific tool when recruiting young doctors and even students for a given specialty.Identifying and describing the group habitus of the doctors within each specialty may furthermore contribute to professional development within the specialty, as it could trigger an increased awareness of the specialty's current and desired future profile.

### Limitations of the Study

It is evident that this kind of study due to its small sample size has limitations in terms of generalization. It is also evident that it provides insight on coherences that might nurture further questioning and exploration more than it offers definitive answers.Credibility was though ensured by the firm understanding of the context obtained through prolonged engagement with specialist training settings.The small number of interviewees might lead to bias by acknowledging and attributing personal features to specialty characteristics. However, the overall objective was to examine whether and how this sociological perspective can contribute to the existing knowledge foundation about choice of specialty, as opposed to documenting the extent of its influence.Additional comparative studies are needed on the subject of specialty choice, using the same conceptual framework (addressing habitus, field, and capital) but also including more specialties. An international perspective would be favorable because transferability is limited when only one national setting is included.

## Conclusions

Bourdieu’s concepts are useful in broadening our under-standing of choice of specialty. A common basis in the form of norms, values, and attitudes shared between senior and junior doctors has an important influence on the choice of specialty. Habitus, which is formed during the doctor's upbringing, influences the choices made later in life, as habitus defines a “space of possibility.” Junior doctors choose a specialty on the basis of who they are: not merely as a doctor, but also as an individual. If the doctor's habitus does not bear some resemblance to the group habitus characterizing the doctors within the specialty, the specialty will not be included in the subset from which the doctor makes his or her choice. As a consequence, junior doctors make their choice from a limited subset of specialties that represent familiar and treasured norms, values, and attitudes, reflecting the doctors' own habitus.

## Appendix 1: The Vignette

Illustrative narrative made on the basis of an interview (2007). From horse enthusiast to gynecologist with a spe-cial interest in cervical cancer: Laura grew up in a safe working class home with no academic aspirations. Her spare time was filled with creative and physical activities like horse rid-ing, surfing, and sewing. She was very aware that she was different because she lived with her grandparents rather than with her parents, who were di-vorced. She was encouraged to do her school work and taught to nurture her talents. In adolescence, she was an elite gymnast for several years. She had a strong desire to help other peo-ple by "serving a purpose and assum-ing social responsibility." After gradu-ating high school with honors, she wanted to make full use of the achieved result, and therefore chose to study medicine. During her studies, she struggled with chemistry, physics, and rote learning; these elements were far from her idea of what being a doc-tor was all about. It was only during her graduate studies, when she started to do research, that she felt sure she had found her niche. Her real choice of specialty was limited to oncology, urology, or gynecology. For Laura, the choice was about sever-al things: "Well, I really liked operating. I still do, actually."

A specific experience with a female patient who was dying from cancer made her decide that she would con-tinue working with cancer. Further-more, during her period as an intern, she held a part-time job at a private humanitarian organization focusing on helping and counselling women (Mødrehjælpen), where she served as an abortion counselor for young wom-en. Here, Laura really felt that she made a difference, and she "took great pleasure in combining the professional, humanitarian, and social aspects of her life." To Laura, the doctors she met from various specialties were important to her choice in a way that transcended the mere technical/surgical aspects of the job. It was also about "whether you perceive the patient the same way, whether you communicate in the same manner with patients and relatives...", and therefore she eventually decided that she felt more at home in the field of gynecology.

## References

[1] The Danish Society of Obstetrics and Gynecology; 2007 [Cited 2010 November 22]. Available from: http:// www.dsog.dk/files/Maalbeskrivelse_gynobs_2007.pdf

[2] The Danish National Board of Health; 2007 [Cited 2010 November 22]. Available from: http://www.sst.dk/Uddannelse%20og%20autorisation/Special%20og%20videreuddannelse/Laege/Maalbeskrivelser%20i%20speciallaegeuddannelsen.aspx

[3] Ranta M Hussain SSM Gardiner Q. Factors that inform the career choice of medical students: implications for otolaryngology. J Laryngol Otol. 2002;116:839-41 12437841 10.1258/00222150260293673

[4] Teitelbaum H Ehrlich N Travis L. Factors affecting specialty choice among osteopathic medical students. Acad Med. 2009;84:718-723 19474544 10.1097/ACM.0b013e3181a43c60

[5] Raphael S Lingard L. Choosing pathology: a qualitative analysis of the changing factors affecting medical career choice. JIAMSE. 2005;15:81-91

[6] Wright S Wong A Newill C. The impact of role models on medical students. J Gen Intern Med. 1997;12:53-56 9034946 10.1046/j.1525-1497.1997.12109.x PMC1497058

[7] Burack JH Irby DM Carline JD Ambrozy DM Ellsbury KE Stritter FT. A study of medical students' specialty-choice pathways: trying on possible selves. Acad Med. 1997;72:534-541 9200589 10.1097/00001888-199706000-00021

[8] Wright S. Examining what residents look for in their role models. Acad Med. 1996;71:290-292 8607931 10.1097/00001888-199603000-00024

[9] Kenny NP Mann KV MacLeod H. Role modeling in doctors' professional formation: reconsidering an essential but untapped educational strategy. Acad Med. 2003;78:1203-1210 14660418 10.1097/00001888-200312000-00002

[10] Mutha S Takayama JI O’Neill EH. Insights into medical students' career choices based on third- and fourth-year students' focus group discussions. Acad Med. 1997;72:635-640 9236475 10.1097/00001888-199707000-00017

[11] Borges NJ Stratton TD Wagner PJ Elam CL. Emotional intelligence and medical specialty choice: findings from three empirical studies. Med Educ. 2009; 43:565-72 19493181 10.1111/j.1365-2923.2009.03371.x

[12] Borges NJ Savickas ML. Personality and medical speciality choice: a literature review and integration. Journal of Career Assessment. 2002;10:362-80

[13] Borges N Savickas ML Jones BJ. Holland’s theory applied to medical speciality. Journal of Career Assessment. 2004;12:188-206

[14] Reed VA Jernstedt GC Reber ES. Understanding and improving medical student specialty choice: a synthesis of literature using Decision Theory as a referent. Teach Learn Med. 2001;13:117-129 11302032 10.1207/S15328015TLM1302_7

[15] Schwartz RW Haley JV Williams C Jarecky RK Strodel WE, Young, et al. The controllable lifestyle factor and students' attitudes about specialty selection. Acad Med. 1990; 65:207-10 2306321 10.1097/00001888-199003000-00016

[16] Lambert TW Davidson JM Evans J Goldacre M. Doctor’s reason for rejecting initial choices of specialties as long-term careers. Med Educ. 2003; 37:312-318 12654115 10.1046/j.1365-2923.2003.01473.x

[17] Thornton J Esposto F. How important are economic factors in choice of medical specialty? Health Econ. 2003;12:67-73 12483762 10.1002/hec.682

[18] Lane CA Healy C Ho M Pearson S Brown MA. How to attract a nephrology trainee: quantitative questionnaire results. Nephrology. 2008;13:116-23 18275499 10.1111/j.1440-1797.2007.00887.x

[19] Grant J, Jones H, Maxted M, Macdonald, Owen H. A review of career decisions and patterns of employment in relation to the characteristics of medical school entrants and medical education. Open University Centre for Education in Medicine; 2002.

[20] Steensen J. On the unacknowledged significance of teacher’s habitus and dispositions. In Bayer M; Brinkjaer U; Plauborg H and Rolls S, ed. Teacher’s career trajectories and work lives. Dordrecht: Springer; 2009.

[21] Bourdieu P. Homo Academicus. Stockholm: Brutus Östlings Bokforlag; 1996.

[22] Bourdieu P & Wacquant LJD. An invitation to reflexive sociology. Cambridge: Polity Press; 1992.

[23] Wilken L, Habitus, kapital og felt - Bourdieus greb til en analyse af praksis. In: Esmark A, Laustsen CB, Andersen NÅ, ed. Socialkonstruktivistiske analysetilgange. Roskilde Universitetsforlag; 2005.

[24] Mahar C, Harker R, Wilkes C. The basic theoretical position. In: Harker R, Mahar C & Wilkes C, ed. Introduction to the work of Pierre Bourdieu. The practice of theory. London: Macmillan; 1990.

[25] Bourdieu P. The forms of capital. In: Halsey A, Lauder P, Brown P, Stuart Wells A. Education, culture, economy, society. Oxford: Oxford University Press; 1997.

[26] Erlandsen DA, Harris EL, Skipper BL, Allen SD. Doing naturalistic inquiry: a guide to methods. California: Sage Publications; 1993.

[27] Bourdieu P, Accardo A, Balazs G, Beaud S, Bonvin F, Bourdieu E et al. The weight of the world. Social suffering in contemporary society. Cambridge: Polity Press. 1999.

[28] Kvale S. Interview: an introduction to qualitative research interviewing. California: Sage Publication; 1996.

[29] Bourdieu P. Distinktjonen. En sociologisk kritikk av dømmekraften. Oslo: Pax Forlag A/S; 1995.

[30] Bourdieu P. The social structures of the economy. Cambridge: Polity Press; 2005.

